# Impact of Three Safety Interventions Targeting Off-Label Use of Immediate-Release Fentanyl on Prescription Trends: Interrupted Time Series Analysis

**DOI:** 10.3389/fphar.2022.815719

**Published:** 2022-04-05

**Authors:** Aníbal García-Sempere, Isabel Hurtado, Salvador Peiró, Francisco Sánchez-Sáez, Clara Liliana Rodríguez-Bernal, Magda Puig-Ferrer, Manuel Escolano, Gabriel Sanfélix-Gimeno

**Affiliations:** ^1^ Foundation for the Promotion of Health and Biomedical Research of Valencia Region, FISABIO, Valencia, Spain; ^2^ Spanish Network for Chronic Health Services Research, REDISSEC, Valencia, Spain; ^3^ General Directorate for Pharmacy, Valencia Health System, Valencia, Spain

**Keywords:** fentanyl, appropriateness of prescription, interrupted time series, policy interventions, pharmacoepidemiology

## Abstract

**Background:** The Spanish health authorities are concerned by the off-label use of immediate-release formulations of fentanyl (IRF) in noncancer pain and cancer pain in patients with no chronic pain therapy.

**Aim:** To evaluate the impact of different interventions to improve appropriateness of IRF prescription on off-label prescription.

**Patients and methods:** We used interrupted time series (ITS) to estimate immediate and trend changes of IRF prescription for noncancer pain (NCP) and breakthrough cancer pain (BCP) in patients with and without chronic cancer pain therapy associated with two medication reviews (I1 and I2) and the issue of a safety warning letter (I3) with data from a Spanish region with 5 million inhabitants, from 2015 to 2018.

**Results:** The use of IRF for NCP in the region Valencia was reduced from about 1,800 prescriptions per week to around 1,400. The first medication review was followed by an immediate level change of −192.66 prescriptions per week (*p* < 0.001) and a downward trend change of −6.75 prescriptions/week (*p* < 0.001), resulting in a post-intervention trend of −1.99 (*p* < 0.001). I2 was associated with a trend change of -23.07 (*p* < 0.001) prescriptions/week. After I3, the trend changed markedly to 27.23 additional prescriptions/week, for a final post-intervention trend of 2.17 (*p* < 0.001). Controlled-ITS provided comparable results. For potentially inappropriate BCP use, the second medication review was followed by a downward, immediate level change of −10.10 prescriptions/week (*p* = 0.011) and a trend change of 2.31 additional prescriptions/week (*p* < 0.001) and the issue of the safety warning (I3) was followed by a downward trend change of −2.09 prescriptions/week (*p* = 0.007).

**Conclusion:** Despite IRF prescription for NCP decreased, the interventions showed modest and temporary effect on off-label prescription. Our results call for a review of the design and implementation of safety interventions addressing inappropriate opioid use.

## Introduction

Although the patterns of use of opioids in Europe are not comparable to the devastating misuse and overuse phenomenon that occurred in the United States in the 2010s, many European countries report an increasing trend in the use of opioids, and some of them are amongst the largest consumers of strong opioids worldwide (Häuser et al., 2021). Among the latter, fentanyl intake has recently seen unprecedented growth and is the most frequently used strong opioid in several countries, including Spain (Hider-Mlynarz et al., 2018; Bosetti et al., 2019; Hurtado et al., 2020; Salazar et al., 2020).

Over the last years, Spanish health authorities have been concerned by the observed prescribing trends of immediate-release formulations of fentanyl (IRF), a drug 80 to 100 times stronger than morphine, which use is associated with a potential high risk of misuse, abuse, addiction, overdose and serious complications (U.S. Food and Drug Administration, 2014; González-Bermejo et al., 2021a). IRF is approved in Spain for use in patients with breakthrough cancer pain (BCP) who are already on chronic treatment for cancer pain (Spanish Agency of Medicines and Medical Devices, 2021a), but there is compelling evidence of its off-label use in noncancer pain (NCP) and in patients with cancer but not on chronic cancer pain treatment. The rate of first prescriptions of IRF in primary care prescribed for NCP in 2016 was 40% (Spanish Agency of Medicines and Medical Devices, 2021b), and over the past few years 60% of reported cases of IRF-related abuse and dependence have been linked to off-label use (González-Bermejo et al., 2021b).

In this context, different interventions have been implemented to improve the appropriateness of IRF prescription. In the region of Valencia, an eastern territory with five million inhabitants, the growing trend of IRF use led the Valencia Health System (VHS) to implement a medication review intervention on 4 January 2016, mandating the regional pharmacy services to case-by-case audit all IRF prescriptions issued in the region for NCP diagnoses, high dose use of IRF and prescription for BCP in patients with no cancer pain maintenance therapy. Pharmacists contacted prescribers individually with the aim to perform a shared assessment of the appropriateness of their prescriptions for IRF in the aforementioned cases, as well as for establishing different therapeutic targets: interruption of IRF, switch to switch to non-opioid analgesic, tapering strategies, or addition to or substitution with pain maintenance therapy in the case of cancer patients (Valencia Regional Governm, 2021). Later, on 16 October 2017, a second medication review intervention was implemented in the region that included as well a case-by-case audit focusing on prescriptions of IRF for NCP but added administrative hurdles and measures for NCP prescribing, such as the need to fulfil additional formularies and to obtain informed consent from the patient in case of continuation of off-label IRF therapy after the review. According to internal documentation of the General Directorate for Pharmacy of the VHS, these two interventions resulted in discontinuation or modification of about a third of IRF treatments in the region during the period they were enforced, but their long-term impact on the volume of prescription remains unknown. Finally, on 21 February 2018, the Spanish Agency of Medicines issued a Safety Warning Letter on IRF use. Warning Letters are informative documents that are disseminated among all the prescribers in the country and are conceived as a reminder of information and to make recommendations. In this case the Letter reminded of the importance of strictly respecting the approved indications of IRF when prescribing and recommended alternatives to IRF treatment in patients with NCP (Spanish Agency of Medicines and Medical Devices, 2021b).

The aim of this study was to assess the impact of the two regional interventions and the national safety warning on the trends of IRF prescription for NCP and BCP in the region of Valencia for the period 2015–2018.

## Methods

### Study Design

In this population-based, quasi-experimental study, we used interrupted time series analyses with data from a 205-week period to evaluate the changes in the number of weekly prescriptions of IRF for NCP and BCP, in patients with or without chronic pain therapy, associated with the implementation of three different interventions targeting non-approved use.

### Setting

The study took place in the region of Valencia (Spain) and, specifically, in the population covered by the public Valencia Health System (VHS), which comprises about 97% of the region’s inhabitants. We included all prescriptions for IRF issued in the region from 1 January 2015, to 30 November 2018. To determine overlapping chronic cancer pain, we also included all the prescriptions of strong opioids issued in the region during that same period indicated for chronic cancer pain control (extended-release formulations of morphine, oxycodone or tapentadol; transdermal fentanyl or buprenorphine, and hydromorphone) prescribed to patients who had at least one IRF prescription for BCP.

### Interventions

We aimed to assess the effect of three interventions on IRF use: a medication review starting on week 53 and lasting for 10 weeks (I1: from 4 January 2016, to 10 March 2016), a second medication review at week 146 and lasting for 6 weeks (I2: from 17 October 2017, to 1 December 2017), and the issue of a Safety Warning letter of the Spanish Agency of Medicines on week 164 (I3).

### Data Sources

Data were obtained from the VHS Integrated Database (VID). VID is the result of the linkage, by means of a single personal identification number, of a set of publicly-owned, population-based healthcare, clinical and administrative electronic databases in Valencia, which has provided comprehensive information of the region’s five million inhabitants since 2008. VID includes sociodemographic and administrative data as well as healthcare information such as diagnoses, procedures, laboratory data, pharmaceutical prescriptions and dispensing (including brand and generic name, formulation, strength, and dosing schedule/regimen), hospitalizations, mortality, healthcare utilization and public health data (García-Sempere et al., 2020).

### Outcomes and Treatment Characterization

We evaluated the impact of three interventions on the trend of weekly prescriptions of IRF. IRF prescriptions were allocated to weeks based on the prescription date. We classified IRF prescriptions into NCP or BCP based on the indication associated with each prescription, using ICD-9 codes and the classification for types of pain proposed by Zhu et al. (2019). For NCP, we further stratified prescriptions into chronic NCP and acute NCP. A marginal proportion of prescriptions (n: 1,140, 0.05% of the total volume) was not associated with a pain diagnose and was excluded from analyses. For prescriptions associated with BCP indications, we determined the presence or absence of chronic treatment for cancer pain by checking at the individual-patient level whether the date of the prescription of IRF was also covered by opioid chronic pain control medication. Days covered with chronic cancer pain medication were estimated using the dosing and regimen scheduled in each prescription.

### Statistical Analysis

First, we constructed weekly series of IRF prescriptions for NCP and for BCP in patients with and without chronic cancer pain therapy. Second, we used interrupted time series (ITS) and segmented linear regression models to assess changes in IRF utilization for NCP while controlling for previous levels and trends after the three intervention dates, and we further stratified the analyses for chronic NCP and acute NCP. Third, in order to control for potential confounding and to contrast the results of the previous analysis, we employed controlled-ITS, using the weekly series of prescriptions of IRF for BCP in patients receiving chronic cancer therapy as a control group. This group fulfils the key features for a control group, as it is expected to be unaffected by the intervention (appropriate care) and to share potential confounders with the intervention series (Bottomley et al., 2019). Fourth, we evaluated the impact of the interventions on the trends of BCP prescription in patients with and without chronic cancer pain treatment. For all analyses, we first ran ordinary least squares regressions, but the Durbin Watson (DW) test identified serial autocorrelation in the residuals. To account for it, we then used Prais–Winsten regression models and the corresponding DW test (Kobayashi, 1985; Huitema, 2011). We finally estimated the post-intervention trend statistic. All analyses were performed using Stata14 (StataCorp LP, College Station, TX).

## Results

### Noncancer Pain

**FIGURE 1:**
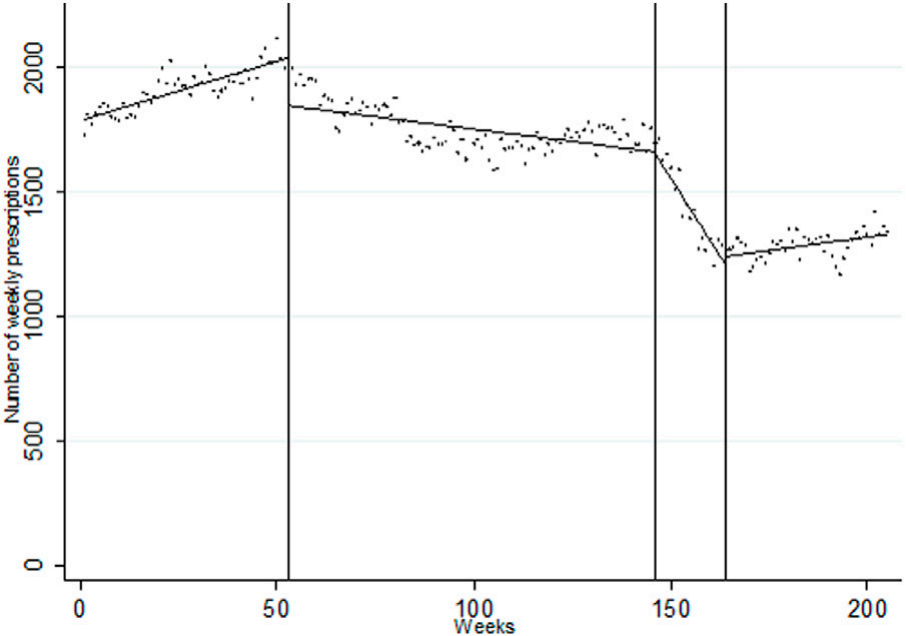
Segmented linear regression trends of weekly series of IRF prescription for NCP, 2015–2018. Dots: observed weekly volume of prescription; Lines: predicted weekly volume of prescription. Week 0: first week of January 2015; Week 53: first medication review; Week 146: second medication review: Week 164: issue of safety warning. Week 205: last week of November 2018. Created by the authors.

**FIGURE 2:**
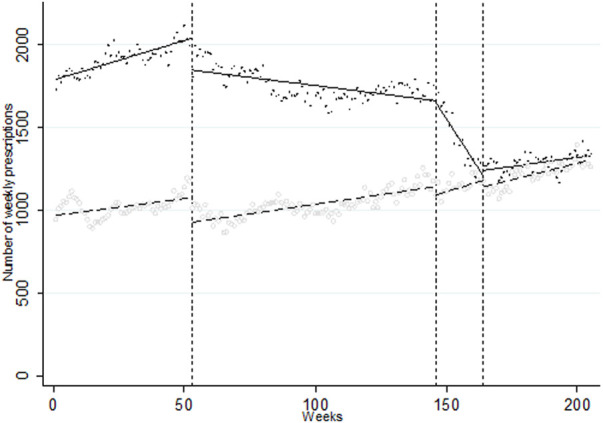
Segmented linear regression trends of weekly series of IRF prescription for NCP using the weekly series of IRF prescription for BCP in patients with chronic cancer pain treatment as a control group, 2015–2018. Crosses/circles: observed weekly volume of prescription for NCP and BCP, respectively; Line/dotted line: predicted weekly volume of prescription for NCP and BCP, respectively. Week 0: first week of January 2015; Week 53: first medication review; Week 146: second medication review: Week 164: issue of safety warning. Week 205: last week of November 2018. Created by the authors.

A total of 342,595 IRF prescriptions for NCP were issued in the period. From an initial constant of 1,791 weekly prescriptions, an upward trend of 4.75 additional prescriptions per week was observed until I1, when an immediate level change of −192.66 prescriptions per week (*p* < 0.001) and a downward trend change of −6.75 prescriptions/week occurred (*p* < 0.001), resulting in a post-intervention trend of −1.99 (*p* < 0.001). I2 was associated with a trend change of −23.07 (*p* < 0.001) leading to a downward post-intervention trend of −29.06 prescriptions/week (*p* < 0.001). After I3, the trend changed markedly to 27.23 additional prescriptions/week, for a final post-intervention trend of 2.17 (*p* < 0.001; see Figure 1 and Table 1). In controlled-ITS using IRF prescription in BCP in patients with chronic pain therapy series as a control group, relative intervention effects were comparable, with a downward relative trend initiating after I1 (−7.01, *p* < 0.001), accentuated after I2 (−25.14, *p* < 0.001) and a change upwards after I3 (27.64, *p* < 0.001; see Figure 2 and Table 1). When stratifying the analyses by chronic (282,628 prescriptions, or 82.5% of NCP prescription) and acute (59,967 prescriptions) noncancer pain treatment we obtained similar results in terms of directionality and intensity (see Supplementary Material S1).

**TABLE 1:** Segmented regression parameters for IRF use for NCP and NCP with a control group.

NCP	Coef.	Std. Err.	t	P>|t|	[95% conf	. Interval]
Prev. slope	4.76	0.85	5.57	0.000	3.07	6.44
I1 (week 53)						
Level change	−192.66	31.62	−6.09	0	−255.01	−130.30
Slope change	−6.75	1.03	−6.052	0.000	−8.79	−4.71
PI trend	−1.99	0.45	−4.43	0.000	−2.88	−1.10
I2 (week 146)						
Level change	−5.21	31.25	−0,0.7	0.870	−66.83	56.41
Slope change	−23.07	3.74	−6.17	0.000	−30.45	−15.69
PI trend	−25.06	3.63	−6.91	0.000	−32.22	−17.91
I3 (week 164)						
Level change	36.28	44.69	0.81	0.418	−51.85	124.41
Slope change	27.23	4.14	6.58	0.000	19.07	35.40
PI trend	2.17	1.07	2.02	0.044	0.056	4.28
Constant	1791.11	24.39	73.42	0.000	1743.00	1839.22
**NCP vs. BCPa**	**Coef.**	**Std. Err.**	**t**	**P>|t|**	**[95% Conf**	**. Interval]**
Prev. slope	2.72	1.95	2.2	0.023	0.37	5.07
I1 (week 53)						
Level change	−44.39	41.16	−1	0.281	−125.31	36.53
Slope change	−7.01	1.45	−4.8	0.000	−9.87	−4.16
PI tren diff.	−4.29	0.62	6.94	0.000	−5.51	−3.08
I2 (week 146)						
Level change	39.14	45.79	0.80	0.393	−50.88	129.17
Slope change	−25.14	4.91	−5.10	0.000	−34.80	−15.48
PI trend diff.	−29.43	4.73	−6.23	0.000	−38.72	−20.15
I3 (week 164)						
Level change	70.93	56.39	1.20	0.209	−39.93	181.78
Slope change	27.64	5.48	5.00	0.000	16.86	38.42
PI tren diff.	−1.79	1.67	−1.07	0.286	−5.08	1.50
Constant	968.60	22.82	42.40	0.000	923.74	1013.46
**Durbin Watson statistic**						
NCP	2.315650					
NCP vs. BCPa	2.316795					

NCP, noncancer pain; BCPa, breakthrough cancer pain in patients with overlapping chronic pain therapy (appropriate); I1,I2, I3, first medication review, second medication review and national safety warning, respectively; PI trend, post-intervention trend; PI trend diff., difference in post-intervention trends between the intervention and control groups.

Created by the authors.

### Breakthrough Cancer Pain

**FIGURE 3:**
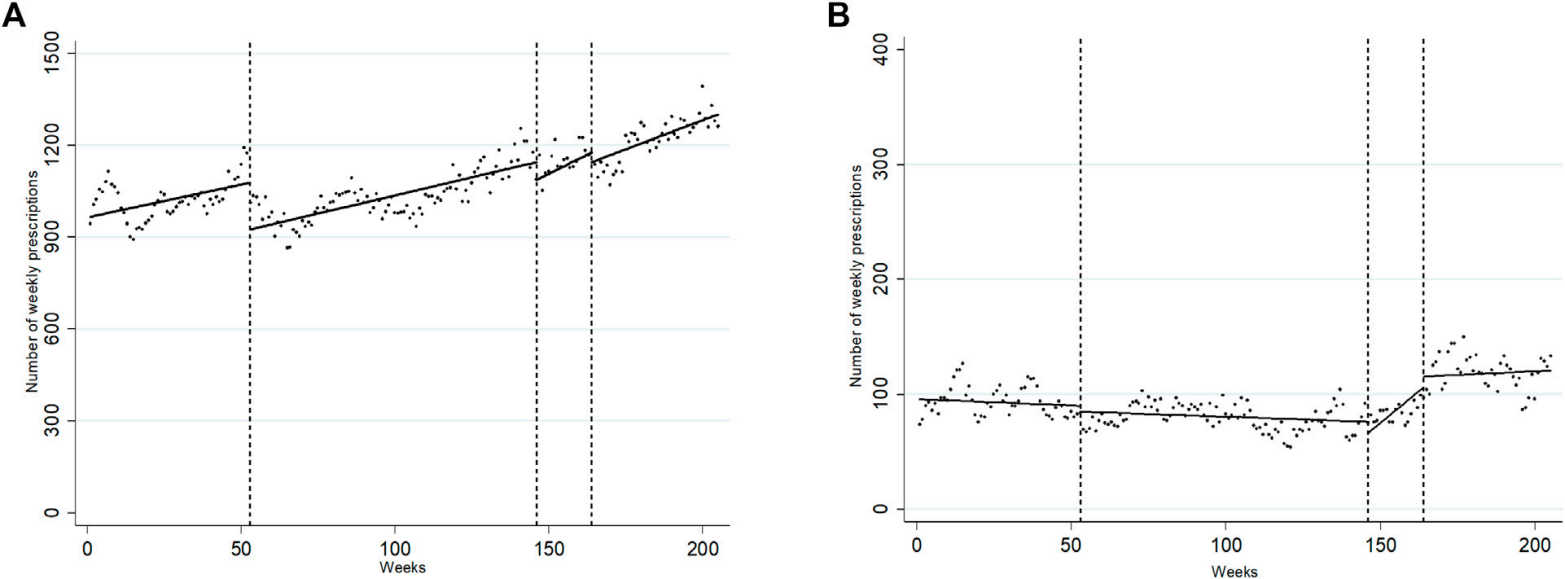
Segmented linear regression trends of weekly series of IRF prescription for BCP in patients with chronic cancer pain treatment **(A)** and in patients without chronic cancer pain treatment **(B)**, 2015–2018. Dots: observed weekly volume of prescription; Line: predicted weekly volume of prescription. Week 0: first week of January 2015; Week 53: first medication review; Week 146: second medication review: Week 164: issue of safety warning. Week 205: last week of November 2018. Created by the authors.

A total of 246150 IRF prescriptions for cancer pain were issued in the period, of which 19,418 (7.89%) were prescribed in the absence of chronic cancer pain therapy. The trend of prescription of IRF for BCP in patients on chronic treatment for cancer pain rose throughout the period and was unaffected by the interventions, even if I1 was associated with a significant, immediate downward level change (−152.26, *p* < 0.001; see Figure 3A and Table 2). With regard to the prescription of IRF for BCP in patients with no overlapping chronic therapy, we found that from an initial constant of 95.70 prescriptions per week, the weekly series of IRF prescriptions was relatively stable until I2. I2 was followed by a downward, immediate level change of −10.10 prescriptions/week (*p* = 0.011) and a trend change of 2.31 additional prescriptions/week (*p* < 0.001). The issue of the safety warning (I3) was followed by a downward trend change of −2.09 prescriptions/week (*p* = 0.007; see Figure 3B and Table 2).

**TABLE 2:** Segmented regression parameters for IRF use for BCP with and without overlapping chronic cancer pain therapy.

Breakthrough cancer pain appropriate	Coef.	Std. Err.	t	P>|t|	[95% conf	. Interval]
Prev. slope	2.13	0.80	2.67	0.008	0.55	3.70
I1 (week 53)						
Level change	−152.26	25.18	−6.05	0.000	−201.93	−102.60
Slope change	0.23	1.00	0.23	0.822	−1.75	2.20
PI trend	2.35	0.44	5.34	0.000	1.48	3.25
I2 (week 146)						
Level change	−57.82	32.25	−1.79	0.075	−121.41	5.78
Slope change	2.63	3.08	0.86	0.393	−3.44	8.71
PI trend	4.989.151	2.890.009	1.73	0.086	−0.71	10.69
I3 (week 164)						
Level change	−31.98	32.95	−0.97	0.333	−96.96	33.01
Slope change	−1.18	3.50	−0.34	0.737	−8.07	5.72
PI trend	3.81	1.34	2.85	0.005	1.18	6.45
Constant	967.00	24.16	40.02	0.000	919.35	1014.64
**Breakthrough Cancer Pain inappropriate**	**Coef.**	**Std. Err.**	**t**	**P>|t|**	**[95% Conf**	**. Interval]**
Prev. slope	−0.11	0.21	−0.52	0.602	−0.53	0.31
I1 (week 53)						
Level change	−5.06	5.07	−1.00	0.319	−15.06	4.93
Slope change	0.02	0.24	0.07	0.942	−0.46	0.50
PI trend	−0.09	0.08	−1.17	0.242	−0.25	0.06
I2 (week 146)						
Level change	−10.10	3.91	−2.58	0.011	−17.82	−2.39
Slope change	2.32	0.64	3.63	0.000	1.06	3.57
PI trend	2.22	0.61	3.64	0.000	1.02	3.43
I3 (week 164)						
Level change	9.22	12.27	0.75	0.453	−14.97	33.41
Slope change	−2.09	0.77	−2.71	0.007	−3.61	−0.57
PI trend	0.13	0.40	0.33	0.740	−0.65	0.91
Constant	95.71	7.79	12.28	0.000	80.34	111.07
**Durbin Watson statistic**						
BCPa	2.137567					
BCPi	2.295185					

BCPi, potentially inappropriate breakthrough cancer pain in patients without overlapping chronic cancer pain therapy; BCPa, breakthrough cancer pain in patients with overlapping chronic pain therapy (appropriate); I1,I2, I3, first medication review, second medication review and national safety warning, respectively; PI trend, post-intervention trend.

Created by the authors.

## Discussion

The use of IRF for NCP in the region Valencia was reduced from about 1,800 prescription per week to around 1,400 in the period 2015–2018. The two regional medication reviews impacted downwards prescription trends, whereas the issue of the national safety warning was followed by a shift towards an increase of IRF prescription. Regarding use for BCP, the interventions did not seem to affect trends or only very marginally. The use of IRF for BCP in patients with chronic cancer pain therapy increased from about 1000 prescriptions per week to levels similar to those of NCP use at the end of the period.

Even though the volume of IRF prescription for NCP decreased, the interventions showed modest or temporary effect on prescription trends. The first medication review changed successfully long-term trends of IRF use for NCP, but the rate of decline of prescription post-intervention was limited. On the other hand, the second intervention, which incorporated additional hurdles to off-label prescribing, achieved a steadier reduction of prescription that lasted only while the intervention was implemented. Shortly after the end of the intervention, a gradual recovery of the number of prescriptions can be observed. In this sense, the drastic trend change after the issue of the Safety Warning detected by ITS modelling should be interpreted with caution, as it probably speaks more of a combination of the lack of impact of the Safety Warning and a *rebound effect* in prescription happening as a response of the ending of the intensive medication review intervention, than of an effect attributable to the national warning. These findings highlight the need to carefully consider potential trade-offs between the intensity and design of interventions and their impact on short and long-term prescribing behaviour.

The volume of use of IRF for BCP in patients without chronic cancer pain treatment was relatively stable in the period, and the interventions showed no or only small effect on prescription trends. In this sense, our data show that a small but potentially concerning use of IRF in these patients in a non-approved indication is maintained throughout the period (accounting for 7.89% of the total prescription for BCP) and is barely affected by the interventions. On the other hand, prescription for BCP in patients with chronic cancer pain treatment followed an ascending trend thought the period. Even if this constitutes appropriate care in regulatory terms, this pattern of growth certainly calls for further attention. Immediate change levels for BCP use were observed after some of the interventions, but these should be interpreted with caution in terms of attribution due to several reasons. For instance, in the case of medication reviews, these are implemented gradually in the territory, and thus abrupt weekly changes should hardly be interpreted as a consequence of the interventions. Also, weekly series can be affected by unmeasured factors (such as holidays, or pharmacy accounting practices) that could translate into sudden changes in particular weeks. Finally, the rationale underlying these drug safety interventions should be to sustainedly improve medication use in the long term, therefore trends seem a more suitable outcome to assess their success than immediate level changes.

Many factors may explain the relative ineffectiveness of the interventions under assessment. In our setting, when a Safety Warning for the Spanish Agency for Medicines is issued, the regional health services passively disseminate this information to prescribers, usually *via* written letter or email. This strategy has been proved to be ineffective to improve physician prescribing (Majumdar and Soumerai, 2003). The impact of regulatory safety warnings from regulatory bodies from Europe and the United States as well as for Spain has been evaluated in a few systematic reviews and studies that have provided mixed results, many of them showing no or modest effect. Even if their interpretation is hindered by the heterogeneity of the studies in terms of design, setting, outcomes analysed and methodological quality, there is a common recognition that many, multilevel and contextual factors may be mediating between the issue of warnings and their impact in clinical practice, such as for instance the very nature of the alert and the risks involved, the implementation of warning-related interventions by the health services, promotional activity or media coverage (Carracedo-Martínez and Pía-Morandeira, 2010; CarracedoMartínez and Pía-Morandeira, 2011; Carracedo-Martínez et al., 2012; Dusetzina et al., 2012; Piening et al., 2012; Carracedo-Martínez and Pia-Morandeira, 2016; Goedecke et al., 2018; Hurtado-Navarro et al., 2019; Georgi et al., 2020; Vázquez-Mourelle et al., 2020).

Medication review interventions that rely on active communication (as peer-comparison and audit) may impact prescribing, but only to a modest extent (Majumdar and Soumerai, 2003). In our case, they lead to modification or withdrawal of IRF treatment in about a third of patients treated with IRF. Among other potentially limiting factors such as prescriber unwillingness to comply with medication review recommendations, and despite the risks and controversies surrounding its use, there may still be clinical situations in acute NCP care (or exacerbated chronic NCP) where IRF may be a therapeutic choice to be considered, regardless of regulation. In this way, only partial effect may be reasonably expected. In our case, two apparently very similar medication review interventions seemed to have different impact in terms of intensity and duration. This inconclusive finding is aligned with international evidence on the effectiveness of opioid medication review interventions. In addition to the factors mentioned above, other qualitative and contextual mediating factors may also play an important explanatory role, such as individual commitment, leadership, interpersonal skills, political priority, financial incentives, etc (Gomes et al., 2014; Chang et al., 2016; Chang et al., 2018; Winstanley et al., 2018; Brighthaupt et al., 2019; Ranapurwala et al., 2019; Bhimji et al., 2020; Rao et al., 2020). Even so, multifaceted interventions designed with a strategic continuity over time and pursuing a sustained improvement of prescribing may prove more suitable to enable long-term prescription changes than isolated, one-component, one-off efforts (Majumdar and Soumerai, 2003; Huiskes et al., 2017).

This study has some limitations. First, the VID databases gather real-world clinical practice data and contain information as registered by health professionals during routine clinical practice, but data are not specifically prepared for research. In this sense, studies based on real-world clinical information like VID are at risk of well-known biases such a differential recording, misclassification bias or missing data. However, prescription and dispensation information (the essential data in this study) is of the highest quality, as it is used for billing purposes, and it includes paperless electronic prescription, the registration of any dispensation in any community pharmacy, and reimbursement to pharmacies in a traceable way for each pharmaceutical package and each patient. Second, although our analytic approach is considered one of the strongest non-experimental approaches for evaluating time-delimited interventions, we cannot rule out the possibility that the changes we observed were due to other events that occurred simultaneously with the interventions. However, we do not know of any other regional or national policies over the observation period that could have affected our results. Third, VID does not include data of inpatient medication, although consumption of IRF in this setting is expected to be marginal. Fourth, we did not employ a minimum daily-dosing criterion to define chronic cancer pain treatment, which could result in a slight mislabelling bias. Also, we only required 1 day of overlap to define appropriate IRF BCP prescription, which could result in a slight overestimation of appropriate prescription. However, this definition is commonly employed to define overlap in opioid-related real-world studies. Fifth, we did not investigate whether the interventions had an impact on the use of alternative treatments such as fast-release formulations of tapentadol or oxycodone. Sixth, we did not investigate whether the interventions had impact on the number of patients treated (see Supplementary Material S2), or on relevant clinical outcomes such as mortality, overdose or addiction. Finally, the generalization of our results to other settings outside Spain, or even to other Spanish regions, should be approached with great caution as contextual factors may play an important role in prescription patterns.

Our results call for a review of the design and implementation of policy interventions addressing IRF prescription quality. Even if the interventions showed modest, temporary, and uneven impact on prescription trends in noncancer pain, prescription of IRF decreased in the period 2015–2018. However, many signs indicate that the problem is far to be resolved. First, the increase of use in cancer pain and the sustained, potentially inappropriate use in a small group of patients with cancer call for attention. Second, in 2021 the country is the third largest consumer of fentanyl worldwide, only surpassed by Germany and the United States (International Narcotics C, 2021), and Valencia is the top consumer region of fentanyl in the country (among 17 regions, with a range of 3.80–1.70 DHD) (Ficheros de facturación and y Mutualidades., 2020) In this context and by the end of 2020, the VHS implemented a third medication review targeting IRF prescription, with a focus on reducing NCP prescription and long-term use of IRF for BCP, which entailed a modification of the electronic prescription system which now compels the prescriber to specify whether the prescription of IRF is “off-label.” Finally, at a national level, a prior authorization scheme for IRF prescription entered into force in the country, 2021, by which every prescription gets to be validated by a so-called medical inspector before it is accepted for public funding and dispensing. In a context of contrasting reactions for and against the measure by primary care pharmacists (Prior Authorization Scheme for Immediate-Release Fentanyl: Measures for an Uncontrolled Problem, 2021), patient associations and pain societies (Letter to the Ministry of Health about the Prior Authorization Scheme for Immediate-Release Fentanyl, 2021), these two latter interventions and their effect on the trends of use of IRF and related outcomes warrant further investigation.

## Access to Data

Legal restrictions on sharing the data set apply as regulated by the Valencia regional government by means of legal resolution by the Valencia Health Agency (2009/13312) which forbids the cession of data to third parties (accessible at: http://www.san.gva.es/documents/152919/157920/resolucionsolicituddatos.pdf). Upon request, authors can allow access to the databases in order to verify the accuracy of the analysis or the reproducibility of the study. Requests to access the datasets should be directed to Management Office of the Data Commission in the Valencia Health Agency (email: solicitud_datos@gva.es; telephone numbers: +34 961-928207; +34 961-928198).

## Data Availability

The datasets presented in this article are not readily available because legal restrictions on sharing the data set apply as regulated by the Valencia regional government by means of legal resolution by the Valencia Health Agency [2009/13312] which forbids the dissemination of data to third parties (accessible at: http://www.san.gva.es/documents/152919/157920/resolucionsolicituddatos.pdf). Upon request, authors can allow access to the databases in order to verify the accuracy of the analysis or the reproducibility of the study. Requests to access the datasets should be directed to Management Office of the Data Commission in the Valencia Health Agency (email: solicitud_datos@gva.es; telephone numbers: +34 961-928207; +34 961-928198) Requests to access the datasets should be directed to “solicitud_datos@gva.es”.
