# Supplementary material for: Impact of Three Safety Interventions Targeting Off-Label Use of Immediate-Release Fentanyl on Prescription Trends: Interrupted Time Series Analysis
Source: Front Pharmacol. 2022 Apr 5;13:815719. doi: 10.3389/fphar.2022.815719 (PMC9016332; doi:10.3389/fphar.2022.815719)
Supplement: Supplementary file 1 [file DataSheet1.docx]

**Impact of Three Safety Interventions Targeting Off-label Use of Immediate-Release Fentanyl on Prescription Trends: Interrupted Time Series Analysis**

**Supplemental Material**

**S1. Results for Chronic and Acute Noncancer Pain**

***Chronic Noncancer Pain***

|  |  |  |  |  |  |  |
| --- | --- | --- | --- | --- | --- | --- |
|  |  |  |  |  |  |  |
|  | Coef. | Std. Err. | t | P>\|t\| | [95% Conf. | Interval] |
| Previous trend | 3,286595 | 0,8441879 | 3,89 | 0 | 1,62179 | 4,9514 |
| Level change w53 | -177,8014 | 29,97163 | -5,93 | 0 | -236,9078 | -118,695 |
| Trend change w53 | -5,103177 | 0,9737537 | -5,24 | 0 | -7,023496 | -3,182858 |
| Level change w146 | 3,738953 | 29,55123 | 0,13 | 0,899 | -54,53841 | 62,01632 |
| Trend change w146 | -15,73752 | 2,748786 | -5,73 | 0 | -21,15834 | -10,3167 |
| Level change w164 | 25,59781 | 32,39193 | 0,79 | 0,43 | -38,28164 | 89,47726 |
| Trend change w164 | 18,94263 | 3,047806 | 6,22 | 0 | 12,93211 | 24,95314 |
| Constant | 1515,222 | 23,60188 | 64,2 | 0 | 1468,678 | 1561,767 |
|  |  |  |  |  |  |  |
|  |  |  |  |  |  |  |

*Dots: observed; Line: predicted. Week 0: first week of January, 2015; Week 53: first medication review; Week 146: second medication review: Week 164: issue of safety warning. Week 205: last week of November, 2018*

***Acute Noncancer Pain***

|  |  |  |  |  |  |  |
| --- | --- | --- | --- | --- | --- | --- |
|  |  |  |  |  |  |  |
| rec | Coef. | Std. Err. | t | P>\|t\| | [95% Conf. | Interval] |
| Previous trend | 1,486668 | 0,2920634 | 5,09 | 0 | 0,9106956 | 2,06264 |
| Level change w53 | -16,4279 | 10,80157 | -1,52 | 0,13 | -37,72946 | 4,873656 |
| Trend change w53 | -1,663854 | 0,3236323 | -5,14 | 0 | -2,302082 | -1,025625 |
| Level change w146 | -2,912459 | 7,794148 | -0,37 | 0,709 | -18,28313 | 12,45822 |
| Trend change w146 | -7,811713 | 1,33392 | -5,86 | 0 | -10,44231 | -5,181117 |
| Level change w164 | 14,08741 | 16,53994 | 0,85 | 0,395 | -18,53067 | 46,70548 |
| Trend change w164 | 8,767243 | 1,49113 | 5,88 | 0 | 5,826617 | 11,70787 |
| Constant | 276,255 | 8,851635 | 31,21 | 0 | 258,7989 | 293,7112 |
|  |  |  |  |  |  |  |
|  |  |  |  |  |  |  |

*Dots: observed; Line: predicted. Week 0: first week of January, 2015; Week 53: first medication review; Week 146: second medication review: Week 164: issue of safety warning. Week 205: last week of November, 2018*

S2. Yearly number of patients with at least one prescription for NCP and BCP (2018 data available up to November 31, 2018)

|  | Chronic NCP | Acute NCP | BCP with chronic cancer pain treatment | BCP without chronic cancer pain treatment |
| --- | --- | --- | --- | --- |
| 2015 | 4,780 | 1,431 | 3,684 | 599 |
| 2016 | 4,855 | 1,646 | 4,014 | 685 |
| 2017 | 4,865 | 1,695 | 4,211 | 692 |
| 2018 | 3,749 | 982 | 4,687 | 727 |
